# Clinical Translation of Combined MAPK and Autophagy Inhibition in *RAS* Mutant Cancer

**DOI:** 10.3390/ijms222212402

**Published:** 2021-11-17

**Authors:** Jennifer J. Lee, Vaibhav Jain, Ravi K. Amaravadi

**Affiliations:** Abramson Cancer Center, Department of Medicine, University of Pennsylvania, Philadelphia, PA 19104, USA; jeesoole@sas.upenn.edu (J.J.L.); Vaibhav.Jain@Pennmedicine.upenn.edu (V.J.)

**Keywords:** *RAS*, autophagy, lysosome

## Abstract

*RAS* (rat sarcoma virus) mutant cancers remain difficult to treat despite the advances in targeted therapy and immunotherapy. Targeted therapies against the components of mitogen-activated protein kinase (MAPK) pathways, including *RAS*, RAF, MEK, and ERK, have demonstrated activity in *BRAF* mutant and, in limited cases, *RAS* mutant cancer. *RAS* mutant cancers have been found to activate adaptive resistance mechanisms such as autophagy during MAPK inhibition. Here, we review the recent clinically relevant advances in the development of the MAPK pathway and autophagy inhibitors and focus on their application to *RAS* mutant cancers. We provide analysis of the preclinical rationale for combining the MAPK pathway and autophagy and highlight the most recent clinical trials that have been launched to capitalize on this potentially synthetic lethal approach to cancer therapy.

## 1. Introduction

Activating mutations in the *RAS* (rat sarcoma virus) oncogene have been studied for decades because of their prevalence in cancer, and because *RAS* mutant cancers have remained difficult to treat. *RAS* is a family of GTPase proteins that are involved in cell proliferation and differentiation [1]. There are three *RAS* homologs in humans: *HRAS*, *NRAS*, and *KRAS*. While *KRAS* mutations are more prevalent in adenocarcinoma, *NRAS* mutations are more prevalent in melanomas, thyroid cancers, and leukemias [2,3]. In addition to the tissue-specific expression of *RAS* homologs, there can also be the expression of multiple isoforms of *RAS*, such as *KRAS4A* and *KRAS4B*, in the same tissue [4]. *RAS* proteins have a complex biology that was extensively reviewed recently by Mukhopadhyay et al. [5,6].

In normal cells, *RAS* gets activated through receptor tyrosine kinase (RTK) signaling in response to growth factors. The Cancer Genome Atlas (TCGA) shows that 46% of tumors involve genetic mutations in RTK-RAS signaling that lead to *RAS* activation [7]. Past reports have stated that mutations in *RAS* genes exist in up to 30% of total cancer cases. However, in 2020, an analysis of the somatic mutation incidence across cancers using multiple large databases found that 19% of all types of cancer patients (~3 million cases per year) have *RAS* mutations [8]. Therefore, it is critical to understand *RAS*, its related pathways, and targeting strategies in cancer.

*RAS* mutations most commonly involve gain-of-function missense mutations in codons 12, 13, and 61 [9]. These mutations have been found to protect the GTP-bound (activated) version of *RAS* proteins (*RAS*-GTP) from the action of the guanine nucleotide exchange factors (GEFs) and GTPase-activating proteins (GAPs), which would convert *RAS*-GTP to *RAS*-GDP. The sustained presence of *RAS*-GTP is the primary driver for the hyperactivation of oncogenic signaling [9]. *RAS* mutation induces a change in the affinity of *RAS* proteins for effector proteins in the downstream mitogenic and growth pathways, contributing to uncontrolled cell proliferation [9]. Therefore, targeting *RAS* is a theoretically promising avenue. However, the challenge with targeting *RAS* is its high affinity for guanine nucleotides, limiting the development of a GTP-mimetic, and the lack of suitable binding sites for therapeutic ligands [10].

To overcome these challenges, approaches that directly or indirectly target *RAS* proteins have been explored. Direct approaches involve the allosteric inhibition of *RAS* or targeting post-translational modifications of *RAS* proteins. Indirect approaches to target *RAS* involve inhibiting the downstream pathways that *RAS* mutant cancers critically rely on to survive and proliferate. The activated *RAS* activates several signaling pathways, but the main two are the mitogen-activated protein kinase (MAPK) and phosphoinositide-3 kinase (PI3K) pathways [11]. In the MAPK pathway, *RAS* induces the phosphorylation of RAF, which phosphorylates MEK, which then phosphorylates ERK. Phosphorylated ERK is the key effector protein of the MAPK pathway, which drives cell proliferation and survival; *RAS*-dependent PI3K signaling leads to the phosphorylation of AKT and of downstream mTOR (mechanistic target of rapamycin), which regulates nutrient metabolism and mRNA translation. The MAPK and PI3K signaling converge and regulate autophagy, a cell-intrinsic stress response pathway that promotes survival in cancer cells. This review will highlight new developments in the direct and indirect targeting of *RAS* and focus on combining MAPK and autophagy inhibition as a new strategy in cancer.

## 2. Allosteric *RAS* Inhibitors

RAS proteins have been described as “undruggable” because the affinity for GTP- GDP is so high that small molecules cannot effectively inhibit the active site. Moore et al. have discussed various ways to directly target *RAS*, which includes an allele-specific covalent inhibition of the GTP binding site [10]. In mice, the deletion of *KRAS* has been found to be more lethal than the deletion of *NRAS* and *HRAS*; therefore, the use of mutant allele-specific inhibitors in humans was proposed to be a more targeted, viable approach for treating *RAS* mutant cancers [10,12,13]. Following decades of iteration, a major breakthrough came with the development of sotorasib, a small molecule that irreversibly binds to KRAS^G12C^ proteins. Sotorasib irreversibly binds to KRAS^G12C^ and prevents the nucleotide exchange required for *RAS* activation, and preclinical studies have found that sotorasib effectively abrogates downstream ERK activity [14]. Convincing preclinical in vivo studies [15,16,17] have led to a phase I clinical trial in patients with *KRAS*^G12C^ solid tumors. Sotorasib produced a 32.2% response rate and a median progression-free survival of 6.3 months and 4.0 months for non-small cell lung cancer (NSCLC) patients and colorectal cancer patients, respectively [14]. While there was inconsistent tumor reduction, the duration of the progression-free survival that sotorasib provided in responders was promising. Currently, there are clinical trials combining sotorasib with other anticancer therapeutics, including trametinib (NCT04185883; NCT04303780). There are other allosteric *RAS* inhibitors such as JAB-21822 entering clinical trials (NCT05002270; NCT05009329) (Table 1). Nearly 2/3 of patients with G12C mutations do not respond to sotorasib, and among those who do respond, some eventually develop cancer progression, suggesting that there are innate and acquired resistance mechanisms to direct *RAS* inhibition. Therefore, additional approaches are needed despite this major advance.

## 3. Inhibition of Post Translational Modification of *RAS*

*RAS* activation requires post-translational fatty acid modification, including farnesylation, geranylgeranylation, and palmitoylation [18]. These fatty acid modifications regulate the membrane association and subcellular localization of *RAS*. Early attempts to target *RAS* in cancer patients focused on the post-translational modification of *RAS*. Farnesyl transferase inhibitors (FTI) and geranylgeranyl transferase inhibitors have been tested in *RAS* mutant cancers with FTIs, such as lonafarnib and tipifarnib, having better drug-like properties and being continued for development in *RAS* mutant cancers [19,20,21].

The enthusiasm for FTIs was tempered when it was discovered that in *NRAS* and *KRAS* mutant cancers, the MAPK pathway activation and tumor growth were not significantly suppressed with FTIs [19,22,23]. Though preventing *RAS* from membrane association was the right approach, inhibiting farnesylation allowed for *NRAS* and *KRAS* mutant cancers to initiate alternative membrane association mechanisms, such as geranylgeranylation, another form of prenylation [24]. The dual inhibition of both farnesylation and geranylgeranylation acts as a way to overcome the activation of alternative prenylation pathways; however, toxicity became dose-limiting [24]. There are, however, some active clinical trials that continue to examine single inhibitor FTI treatments for cancer (NCT04284774; NCT03496766; NCT04865159). These trials are restricted to *HRAS* mutant cancers, where the sole prenylation method is via farnesyltransferase, preventing the circumvention of FTIs with different compensatory prenylation methods in other cancers [10]. The palmitoylation of *RAS* is also an attractive target for drug development [25,26], but to date no *RAS* palmitoylation inhibitor has been developed for clinical trials.

## 4. *RAF* Inhibitors

RAF inhibitors have also been considered as a treatment for cancers with *RAS* mutations; however, it has been found that the inhibition of *RAF* in the presence of mutant *RAS* paradoxically activates the MAPK signaling pathway, leading to enhanced tumor growth [27,28,29]. The process is believed to be driven by *RAF* dimerization, the transactivation of mutant *RAS*, and the resumption of ERK1/2 signaling [30]. A novel *RAF* inhibitor, LXH254—which may have activity in tumors with concurrent BRAF and *NRAS* mutations, and in melanoma cell lines that are resistant to BRAF and MEK inhibition—was found to be less active in the *KRAS* mutant cell lines [31]. In the cell lines expressing ARAF, a paradoxical activation of the MAPK pathway was observed [31]. In the *KRAS* mutant cells lacking ARAF, LXH254 exhibited potent cytotoxicity, suggesting that patient selection could be possible to tailor the use of this agent. LXH254 is in phase I and II clinical trials in both BRAF and *RAS* mutant tumors (NCT02607813, NCT02974725). While a number of new inhibitors of *RAF* dimerization and *RAS* transactivation are being developed, the currently approved *RAF* inhibitors should not be used as a single agent in *RAS* mutant cancers [30].

## 5. MEK Inhibitors

MEK is the serine threonine kinase downstream of *RAS* and *RAF* in the MAPK cascade. MEK inhibitors (MEKi) are often effective at limiting the growth of either *RAS* or *RAF* mutant cancers in preclinical models. MEK inhibitors can produce complete ERK inhibition in preclinical models of *RAS* and *RAF* mutant cancers [32]. However, this effective pathway inhibition and antitumor activity does not usually translate into clinical efficacy. While MEK inhibitors have been tested broadly in many *RAS* mutant cancers, clinical trials have demonstrated very little single agent activity. Trametinib is an FDA-approved MEKi that has shown to be successful in treating melanoma with a *BRAF V600E* or *V600K* mutation; however, it is not clinically approved for *RAS* mutant cancers. A clinical trial in 2013 compared trametinib with docetaxel chemotherapy in NSCLC with a *KRAS* mutation and found no significant difference in the progression-free survival of patients when compared to historical controls [33]. Other FDA-approved MEK inhibitors include cobimetinib and binimetinib. Currently, all three MEK inhibitors are not clinically approved for *RAS* mutant cancers, but the off-label use of these agents is fairly common. A preclinical study demonstrated the antitumor activity of binimetinib in 144 melanoma patient-derived cell lines, including patients with the *NRAS* mutation [34]. Furthermore, a phase III clinical trial for binimetinib produced a low response rate but significantly improved the progression-free survival when compared to dacarbazine chemotherapy [35]. The low response rates for MEK inhibitors in the *RAS* mutant cancers have been observed in other clinical trials as well (NCT01986166, NCT01155453). In addition, MEK inhibitors can produce significant cutaneous, ocular, and cardiac toxicity [36,37,38]. Therefore, it is likely that MEK inhibition at clinically achievable doses is activating one or more resistance mechanisms in *RAS* mutant cancers, limiting the therapeutic efficacy.

## 6. ERK Inhibitors

ERK inhibitors (ERKi) have not been studied as widely as *RAF* and MEK inhibitors; however, ERK inhibitors have entered clinical trials in recent years. Recent preclinical research was performed on seven different *RAS* mutant patient-derived xenograft (PDX) models with LY3214996, a competitive ERK1/2 inhibitor [39]. While the MAPK inhibition levels were similar across the models, the actual tumor regression varied, suggesting that potential compensatory resistance mechanisms develop under ERK inhibition [39]. The ERK inhibitor MK-8352 (the oral derivative of tool compound SCH-772984) showed preclinical evidence of reduced ERK phosphorylation, a decrease in cell proliferation, and increased apoptosis in *RAS* mutant models. However, in a phase I clinical trial, MK-8352 produced a 20% response rate in patients with *RAS* mutant tumors, which was not deemed enough to develop it further as a single agent [40]. Another ERKi, ulixternib, is an ATP-competitive ERK1/2 inhibitor, and its phase I/II clinical trial showed an 18% response rate in *NRAS* mutant melanoma patients [41]. A recent review on ERK has discussed how ERK activation, rather than inhibition, could have pro-death effects [42]. While ERK inhibitors remain promising in the eyes of some clinical investigators, there are very few active ERK inhibitor programs. Therefore, for targeting the MAPK pathway in *RAS* mutant tumors, the focus has shifted to understanding MEK inhibitor resistance mechanisms and the combinations to overcome this resistance.

## 7. Resistance to MEK Inhibitors in *RAS* Mutant Cancers

While once considered a promising treatment for all *RAS* and *RAF* mutant cancers, MEKi face the issue of resistance. Mechanistically, in the context of colorectal cancer, MEKi was found to be promoting tumorigenesis and cell proliferation via Wnt signaling and Akt pathway activation [43,44].

The bromodomain and extra-terminal domain (BET) protein family are epigenetic factors that regulate gene expression. These proteins have been found to be overexpressed in *NRAS* mutant melanoma; moreover, a combination of BET and MEK inhibition showed antitumor effects in otherwise MEKi-resistant tumors [45]. It has also been found that *KRAS* mutant pancreatic ductal adenocarcinoma showed resistance to MEK inhibition and activated mTORC1 and mTORC2, which are key effectors of tumor growth [46].

Additionally, other proteins such as SHOC2 and ERBB3 have also been identified as key proteins involved in the resistance pathways to MEKi [47,48]. Kun et al. have further reviewed the various mechanisms of MEK inhibition resistance, which include reactivation of the MAPK pathway, the activation of parallel pathways such as PI3K and STAT, and the rewiring of signaling pathways via different transcription factors [49,50]. In many cases, the combinations mentioned above have been attempted clinically or the drugs targeting these pathways of potential resistance to MEK inhibition have major drawbacks. Recently, autophagy was identified as a resistance mechanism to MEK inhibition, providing a new druggable target for combination therapy development [51,52,53].

## 8. Autophagy as a Resistance Mechanism to MAPK Inhibition

Autophagy is a cellular process that allows for the degradation and recycling of intracellular material. While there is a basal level of autophagy in all mammalian cells, stressed cells have significantly increased levels of autophagy [54]. The first step of autophagy includes the development of double-membrane vesicles called autophagosomes. Autophagosome generation requires a number of key autophagy protein complexes, including the ULK1 and VPS34 complexes, to prepare the membrane. The autophagy protein LC3 (ATG8) is conjugated to lipids in the emerging autophagic membrane. LC3 also serves as a dock for autophagy cargo receptors that deliver cargo (proteins and organelles that are ubiquitinated and targeted for degradation) into the autophagic vesicle as it forms. Once formed, the fusion of autophagosomes with lysosomes allows for the degradation of the cargo and the recycling of nutrients. For a more in-depth review of the autophagy machinery, see [55,56].

In the models of advanced as-driven cancer in which the autophagy genes are knocked out, autophagy was found to promote tumor growth [57,58,59]. The rapid proliferation of cancer cells requires high levels of energy and nutrients; moreover, autophagy is a process that cancer, a stress-inducing entity, can use to break down unnecessary components for tumor growth [60]. In fact, *Ras* mutant cancers have a higher level of basal autophagy than does healthy normal tissue [61]. In a *Kras^V^*^12*D*^*–Lkb1*^−/−^ model of lung cancer, autophagy was found to be required to support the TCA cycle and for nucleotide synthesis [62]. All of the studies mentioned above focus on the cell-autonomous role of autophagy in *Ras* mutant cancer. Studies performed in sophisticated mouse and fly models of *Ras* mutant tumors have also found nontumor cells in the tumor microenvironment of the *Ras* mutant tumors, and in the distant organs such as the liver utilize the increased autophagy that supports tumor growth, and may also play a role in the cachexia often observed in patients with *RAS* mutant cancer [63,64,65,66,67].

Not only does autophagy promote the growth and survival of tumor cells in advanced cancer, in the context of cancer therapy, including MAPK inhibition, it is a well-known adaptive resistance mechanism. Autophagy was initially demonstrated to be a resistance mechanism to *BRAF V600E* mutant melanoma and brain cancer [68,69,70]. More recently, in *RAS* mutant cancers, autophagy was discovered as a key survival mechanism for cancer cells faced with therapeutic stress. Viale et al. used a doxycycline-inducible mutant *Kras G12D* mouse model of PDAC. Although there was rapid tumor regression upon Dox withdrawal (modeling therapeutic Ras inhibition), there were PDAC epithelial cells that were embedded and dormant in fibrotic tissue in the absence of mutant Kras activity. Transcriptomics and metabolomics analysis of these cells revealed that autophagy was induced as a survival mechanism [71]. Similarly, MEK inhibition was found to induce cytoprotective autophagy in *Kras* mutant NSCLC [51]. Further, in *Ras* mutant lung adenocarcinoma cells, increased autophagic flux limited MEK/ERK inhibition-mediated apoptosis, leading to the survival of cancer cells [52]. However, senescent lung adenocarcinoma cells, which lack the ability to produce autolysosomes, could not survive, highlighting the significance of autophagy for resistant survival against MAPK pathway inhibition [52].

Ojha et al. have found that a combination of MEKi and BRAFi induced ERK reactivation, which led to autophagy induction in melanoma. While most of this work focused on *BRAF* melanoma cell lines, MEK inhibition in *NRAS* mutant melanoma cell lines was also found to activate cytoprotective autophagy through ERK reactivation. The investigators identified a novel mechanism of ERK reactivation that required the ER translocation of MAPK components, followed by the exit of ERK, and the phosphorylation of ERK by PERK. Inhibiting ER translocation not only prevented ERK reactivation but also inhibited autophagy and overcame resistance to MAPK inhibition [72].

## 9. Combined MAPK and Autophagy Inhibition Is Synergistic in *RAS* Mutant Cancer

**Figure 1:**
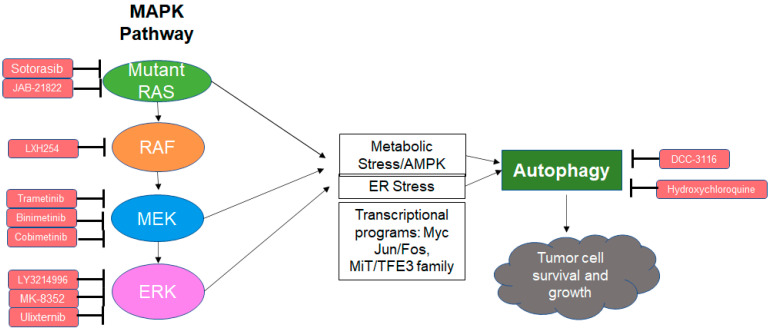
Schematic overview of the MAPK pathway, its inhibitors, and the subsequent autophagy as a resistance mechanism.

Given autophagy’s role in the resistance to MAPK inhibition in *BRAF* mutant tumors, recent attention has turned to the role of autophagy in MAPK therapy resistance in *RAS* mutant cancers (Figure 1). One study involved the knockdown of MAPK and autophagy genes in various iterations in *KRAS* mutant cancer cell lines [73]. The combination of *BRAF*, *CRAF*, and *ATG7* depletion was most successful in reducing cancer cell viability. Moreover, it was found that though *ATG7* depletion alone could not reduce cancer cell viability, this depletion led to heightened sensitivity to the knockdown of *BRAF* and *CRAF* [73]. This corroborative finding characterizes autophagy as a nonessential process for overall cell survival that would improve MAPK inhibition-mediated antitumor activity when inhibited.

While Lee et al. studied the inhibition of *RAF* and autophagy for *RAS* mutant cancers, Bryant et al. examined the inhibition of *RAS*, ERK, and autophagy in PDAC [73,74]. This study used the mCherry-EGFP-LC3B autophagy reporter assay and the bafilomycin clamp assay to demonstrate increased autophagic flux under KRAS suppression in pancreatic cancer cells [75]. Autophagic flux was increased 20-fold following doxycycline withdrawal in a doxycycline-inducible *KRAS G12D*-driven mouse model of PDAC. Associated with this massive upregulation of autophagy was a significant increase in glycolytic activity, suppressed MYC activity, and decreased mitochondrial activity [74]. The authors conclude that autophagy is activated due to metabolic stress induced by the sudden loss of activated KRAS. ERK inhibition using chemical inhibitors produced significantly reduced tricarboxylic acid (TCA) cycle activity; however, autophagy compensated for this loss by providing necessary TCA components. When both ERK and autophagy were inhibited with SCH-772984 and chloroquine or hydroxychloroquine (HCQ), the metabolic crisis characterized by an increase in glycolysis and a decrease of mitochondrial activity continued unchecked, producing synergistic anticancer activity. The combination of SCH-772984 and HCQ once again produces near complete tumor growth impairment of two different human pancreatic PDX tumors, without any weight loss or morbidity in animals, and translating into a significant survival benefit for these mice [74].

In a separate study, Kinsey et al., tested the MEKi and HCQ combination in seven *KRAS*, *NRAS*, and *BRAF* mutant mouse models of multiple cancers. MEK inhibition led to increased autophagic flux and treatment resistance, but the inhibition of autophagy along with the MEK inhibitor drastically reduced the tumor viability [76]. The near complete regression of tumor achieved with trametinib and chloroquine was so uniform and striking that it galvanized efforts to immediately translate the finding into clinical trials (see below). To understand the mechanism at play, immunoblotting for the phosphorylation of downstream autophagy mediators was performed, and the LKB1/AMPK/ULK1 signaling axis was found to be a key autophagy pathway involved. These two recent studies by Bryant et al. and Kinsey et al. highlighted the complexities and the crosstalk of the pathways that relate to MAPK and autophagy; moreover, as existing treatments become refined and new ones develop, it will also be important to identify the most critical pathways for optimized and targeted treatments.

In addition, Kinsey et al. treated a patient with stage IV pancreatic cancer refractory to multiple lines of standard therapy with trametinib and HCQ, producing a partial response (50% tumor shrinkage) and a striking and steep drop in tumor marker CA 19-9 [76]. Other case reports of similar MEKi and HCQ treatment regimens have also supported the tolerability and activity of the regimen. One study found that when two patients with heavily pretreated *KRAS* mutant pancreatic adenocarcinoma were treated with a combination of trametinib and HCQ, both experienced meaningful disease stabilization, although neither experienced a partial response [77]. In another case report, a patient with heavily pretreated metastatic *KRAS* mutant colorectal cancer was treated with binimetinib, HCQ, and the angiogenesis inhibitor bevacizumab. This regimen led to a 17% tumor size reduction and improvement in the clinical symptoms [78]. While these later case reports did not report partial responses with MAPK and autophagy inhibition, the results do support studying these combinations further in highly treatment-refractory patients.

## 10. Clinical Trials with Autophagy and MAPK Inhibitors in *RAS* Mutant Cancers:

There are currently five active clinical trials that involve the combined inhibition of the *RAS*/MAPK pathway and autophagy for patients with *KRAS* mutant cancer (Table 2). These trials include binimetinib and HCQ in pancreatic and NSCLC, and trametinib and HCQ in *KRAS* mutant biliary tract carcinoma and pancreatic cancer. Currently all trials are at either the phase I or II stage. There is also a phase I/II clinical trial that examines *KRAS* mutant pancreatic cancer and combines three treatments: cobimetinib, HCQ, and atezolizumab, a PD-L1 antibody.

There are several other autophagy inhibitors that are under clinical trials for pancreatic, prostate, myeloma, and lymphoma cancers, such as verteporfin and clarithromycin; however, these trials do not combine treatments with a MEKi or other MAPK pathway inhibitors [79]. For autophagy inhibition, HCQ is still being used; however, as novel autophagy inhibitors gain a stronger presence, more potent and specific autophagy inhibitors will be used to overcome resistance mechanisms.

Recently, a ULK1 inhibitor, DCC-3116, entered a first in-human phase I study (NCT04892017). This compound will be dose-escalated as a single agent and then combined with trametinib in *Ras* and *RAF* mutant cancer patients. This is the first example of a non-lysosomal autophagy inhibitor to enter clinical trials, which suggests that other autophagy inhibitor compounds will be entering clinical trials in the coming years. Combinations with MEK and ERK inhibitors are a rational approach for these autophagy inhibitors. Another promising target in the autophagy pathway for which inhibitors are being developed for clinical trials includes vps34.

## 11. Open Questions

Which node of autophagy should be inhibited in combination with MAPK inhibition: lysosomal or non-lysosomal? This remains an open question, but a recent preclinical study has demonstrated that non-lysosomal autophagy inhibition in *KRAS* mutant pancreatic cells upregulates NRF2-dependent micropinocytosis, an alternative nutrient-scavenging pathway that can rescue cell death. Since macropinocytosis is still dependent on lysosomal function, a lysosomal autophagy inhibitor would circumvent this potential Achilles’ heel of autophagy and MAPK inhibition strategy [80]. Which MAPK inhibitor should be used: *RAS*, MEK, or ERK inhibitors? Do novel *RAS* inhibitors induce cytoprotective autophagy, and will the combined *RAS* and autophagy inhibition enhance the efficacy of *RAS* inhibitors in a tolerable manner? It is clear that there are many unanswered questions in this field worthy of further investigation.

## 12. Conclusions

MAPK pathway inhibition alone in *RAS* mutant cancers has been shown to activate compensatory mechanisms and resistance to treatment; therefore, targeting both MAPK and these alternative mechanisms is a promising approach [42,43,44,45,46,47,48,49]. Autophagy, being one of these alternative mechanisms, has become an area of interest for cancer treatment [53,54,55,56,57,58,59,60]. While the relationship between the MAPK pathway and autophagy is not fully understood, there have been recent findings that the mitochondria and metabolic activity, such as AMPK-regulated activity, may be a significant connection between the two [63]. In addition, the activation of the ER stress response as an intermediate pathway that in turn induces autophagy has been proposed [69,72]. It will be important to find the most critical pathways that tie the MAPK pathway and autophagy together, as these findings may allow for more specific, efficient targeting as *RAS* mutant cancer treatment. Most current clinical trials that target both use HCQ as the autophagy inhibitor; however, there are preclinical studies that have nominated more potent autophagy inhibitors for clinical development. Most recently, a ULK1 inhibitor which inhibits autophagic flux in a non-lysosomal manner has entered a clinical trial that will eventually be used in combination with a MEKi in *RAS* and *RAF* mutant cancers (NCT04892017). While there are many open questions in this field, as novel MAPK inhibitors and autophagy inhibitors enter clinical trials, the combined targeting of MAPK signaling and autophagy may demonstrate efficacy in *RAS* mutant cancers.

## Figures and Tables

**Table 1:** Current clinical trials using allele-specific *RAS* inhibitors.

Treatment	Condition	Phase	ClinicalTrials.gov Registration
Sotorasib + Trametinib + Others	Advanced *KRAS G12C* Solid Tumors	I/II	NCT04185883
Sotorasib + Docetaxel	Advanced *KRAS G12C* NSCLC	III	NCT04303780
JAB-21822 + Cetuximab	Advanced *KRAS G12C* NSCLC + CRC	I/II	NCT05002270
JAB-21822	Advanced *KRAS G12C* NSCLC + CRC	I/II	NCT05009329

**Table 2:** Current clinical trials combining MAPK pathway inhibitors and autophagy.

Treatment	Condition	Phase	ClinicalTrials.gov Registration
Binimetinib and HCQ	Stage IV *KRAS* mutant NSCLC	II	NCT04735068
Binimetinib and HCQ	Stage IV Pancreatic adenocarcinoma	I	NCT04132505
Trametinib and HCQ	Stage IV Pancreatic adenocarcinoma	I	NCT03825289
Trametinib and HCQ	Stage IV *KRAS* mutant biliary tract carcinoma	II	NCT04566133
Cobimetinib + Atezolizumab + HCQ	Stage IV *KRAS* mutant gastrointestinal cancers	I/II	NCT04214418

## Data Availability

Not applicable.
